# The Effect of Subjective Perception of Work in Relation to Occupational and Demographic Factors on the Mental Health of Polish Nurses

**DOI:** 10.3389/fpsyt.2020.591957

**Published:** 2020-12-03

**Authors:** Krystyna Kowalczuk, Elżbieta Krajewska-Kułak, Marek Sobolewski

**Affiliations:** ^1^Department of Integrated Medical Care, Medical University of Bialystok, Bialystok, Poland; ^2^Faculty of Management, Rzeszow University of Technology, Rzeszow, Poland

**Keywords:** stress, nurse, mental health, anxiety, depression, insomnia, work characteristics

## Abstract

**Introduction:** Nursing is considered one of the most stressful professions in the world. The high emotional burden associated with excessive workload in qualitative and quantitative terms, exposure to existing and emerging infectious diseases, daily confrontation with the suffering of individuals and their families and low social support leads to the development of numerous stress reactions among nurses, resulting in the development of anxiety, insomnia, social dysfunction and depression. Indeed, somatic and mental stress-related disease rates are higher among nurses than in the general population.

**Aim:** To determine the impact of subjective work characteristics on the mental health of nurses in relation to demographic and occupational factors.

**Material and method:** The research was carried out among 558 nurses working in hospitals in Podlaskie Voivodeship, and used the Subjective Work Evaluation Questionnaire (SWEQ) and Goldberg's GHQ-28 Questionnaire.

**Results:** As measured by SWEQ, and as self-assessed by means of the GHQ-28 questionnaire, *overall stress* negatively affects the nurses' health (*R*^2^ = 18.7%). Among the partial measures of the SWEQ questionnaire, *work overload* had strong and the *lack of rewards, social relations* and *lack of support* had weak negative effect on the overall mental health assessment of nurses (*R*^2^ = 19.2%). The *responsibility* measure was an exception that had a positive impact on the nurses' well-being. Among occupational and demographic factors, only higher education in relation to secondary education in interactions with the *overall stress* measure and *unpleasant work conditions* had a positive effect on the overall mental health self-assessment of nurses (*R*^2^ = 20.7%).

**Conclusions:** The results of our study provide a clear message to the hospital management that improving the work organization and atmosphere of nurses by reducing perceived work overload and increasing the responsibility of nurses can have a positive impact on their mental health. Encouraging nurses to improve their education can result not only in an obvious improvement in staff qualifications, but also in better resistance to stressors in the workplace and, consequently, in better staff well-being. Both measures can have a positive impact on the quality of care provided by nurses and on reducing staff turnover.

## Introduction

The processes of privatization, automation and globalization in the last 20 years in the Polish health service have resulted in significant changes in the working conditions of medical personnel. Workplaces have become safer in terms of exposure to physical and chemical agents reducing the number of employees falling ill with occupational diseases. Concurrently, the pressure on employee efficiency and cost-cutting has become more prevalent. This yields an increase in the number of employees diagnosed with stress-related diseases on annual basis (1).

Physicochemical and psychosocial factors have negative impact on employees' health through stress. In our study, we focused on the analysis of psychosocial factors. They arise under certain organizational and social conditions and their character is determined by the psychological assessment of their significance to the individual - whether they pose a threat, constraint, deprivation of some important values, or a challenge to his or her abilities and aspirations (2). So far, psychosocial factors have generated relatively little interest among occupational medicine services, primarily due to psychosocial stimuli subjective nature (3). Whether a particular element of a work situation can become a psychosocial stressful stimulus is determined by the characteristics of the individual and the group in which the individual works, since the importance of the factor for the employee depends on individual's work enviroment. Psychosocial factors affect employees' health by triggering long-term stress reactions (2). The effects of these reactions may be reflected in disorders of various systems and body functions.

It is difficult to establish a specific link between a specific psychosocial factor and incidence of a specific disease (4). Hence, in our study we decided to investigate psychosocial factors' effect on the overall mental health of employees. The importance of this issue for research results from several indicators: (1) basically all employees are exposed to psychosocial factors, (2) about 25% of all employees complain about excessive stress at work, (3) trends in the development of work processes have brought about an increase in the number of people employed in the service sector, in managerial positions, in operating computer systems and automated devices, (4) economic effects of stress are seen in the form of reduced work quality, increased number of errors and accidents at work, as well as the costs of treating addictions and stress-related diseases (2, 4).

We decided to conduct our study among nurses. Globally, nursing is considered a very stressful profession (5–8). The high emotional burden associated with increasing workload in qualitative and quantitative terms, exposure to existing and emerging infectious diseases (9), the daily confrontation with the suffering of individuals and their families and low social support leads to many stress reactions among nurses (10, 11). Indeed, studies by Allen and Shanock have shown that as they start their first job, young nurses experience enormous mental shock due to low social support and lack of socialization (12).

Society's expectations toward nurses are significantly different from the feelings of nurses themselves (13). The study conducted by Bolton shows that the expectations of society toward the expression of emotions by nurses concern only those emotions that alleviate the fear and suffering of patients and their families. In contrast, personal emotions, the so-called spontaneous, e.g., frustration, loathing, anxiety, should be expressed with a lot of empathy due to the nature of the performed work and the characteristics of the nursing profession (14). Studies by Donoso et al. indicate that the suppression of emotions so as to reduce emotional expression, e.g., due to working conditions, is a considerable stress factor among nurses (15).

The perception of nurses' workload is a subjective feeling, which has been confirmed by professional stress theories. The theory of individual being fitted to the environment, presented by French et al., is built upon on two basic elements: the degree of attitude and ability of the employee to meet the demands of the job. In this theory, there is a distinction between objective reality and subjective perception of changes taking place in the environment. The objective match is based on external established criteria such as experience, education and skills, which are assessed by external experts, e.g., during job interviews. Subjective fitting refers only to the individual characteristics of the employee and his or her personal perception of the working environment. The mismatch may occur in various configurations, each of which may affect the stress felt by the employee (16).

In studies of occupational or work-related stress, a leading theoretical model that is widely used is the Job Demand Control model developed by Karasek (17). This model predicts that workload and subsequent physical or mental illness are the result of the interaction of work requirements and work control. According to the extended Job Demand Control Support model, the highest risk of mental ill health is expected among employees whose jobs are characterized by high demands, low control and low social support (18).

In opposition, the Siegirist model (19) treats the imbalance between work effort and remuneration. Here, the level of effort depends on two main factors: the characteristics of the work (requirements) and specific personal dispositions. The award for work concerns three aspects: financial gratification and professional status, respect and support, as well as job security and career development opportunities. In a situation where there is the excessive involvement of an employee in the work performed, with simultaneous underestimation of the prizes received, there is an imbalance between effort and reward, which leads to a stressful situation (20).

The high social expectations and professional demands placed on nurses by hospital management, juxtaposed to the low salaries constitute a perfect match for testing the aforementioned stress theories. Based on numerous studies, it is known that stressors at work have a negative effect on employee health (21, 22). Somatic and mental stress-related disease rates are higher among nurses than in the general population (2). Indeed, such psychosocial burdens affect the development of anxiety, insomnia, excessive sleepiness and depression among nurses (23). The effects of excessive workloads manifest themselves as undesirable behaviors at work such as avoidance, increased irritability and cynical attitude (2). Any workload, which exceeds the employee's ability to cope is associated with absenteeism, change or resignation from work (24). According to Chen et al., some nurses often take sick leave to avoid the mental strain at work and eventually leave the profession (25). In Poland, for example, a significant percentage of nurses leave the profession within 10 years of obtaining their professional qualifications. Here, the main reasons cited for leaving the profession are low wages, difficult working conditions and poor health (26, 27).

Health condition, sickness-related absenteeism and nurses leaving the profession have a direct impact on the quality of care provided and patients' health results (28). We have hence decided to examine which of the subjective characteristics of work have a significant impact on the overall mental health of the nurses and to what extent demographic and occupational factors influence such relationships. This study is an attempt to fill the gap in the research confirming that improving the organization and working climate of nurses can be a strategic goal of hospital management (29).

## Materials and Methods

The cross-sectional study was conducted in the first 2 weeks of March 2020, in Białystok, in Poland. It included registered nurses working in hospitals and clinics in the Podlaskie Voivodeship. Participation in the study was voluntary, and all procedures were approved by the Bioethical Committee of the Medical University of Bialystok [ref. no APK.002110.2020].

### Study Group Selection

The selection of respondents to the study group was based on the register of associated nurses in the District Chamber of Nurses and Midwives in Bialystok. The total number of registered nurses was 6,085 persons (5,990 women and 95 men). The selection criterion was employment based on employment contract in a hospital. Nurses working part-time and on other than employment contract were ruled out.

### Study Procedure

The applied study was conducted using paper-based questionnaires. The questionnaires were distributed by researchers during trainings organized by the District Chamber of Nurses and Midwives in Bialystok. Participation was voluntary. Before the study, each nurse was informed about the anonymity of the conducted research, and about the possibility of withdrawing from the study without stating a reason. They were asked to fill out the surveys in their free time within 2 weeks and to send the completed questionnaires in a sealed envelope to the investigators' address. There were 800 questionnaire surveys distributed, resulting in 558 correctly completed questionnaires obtained. The response rate was 69%. There are no known reasons why 242 respondents did not participate in the study. All the demographic data was obtained from surveys in the form of respondents' self-reports. No incentives were used to encourage participation in the study.

### Description of the Questionnaire and the Applied Measures

The research tool for health condition assessment was the Goldberg General Health Questionnaire GHQ-28, in the Polish adaptation by Makowska and Merecz (30). The GHQ-28 questionnaire is used to assess the mental health of adults. It allows for the identification of people whose mental condition was subject to temporary or long-term breakdown as a result of experiencing difficulties, problems or as a result of mental illness, and those who are at significant risk of mental health disorders. The GHQ-28 questionnaire, in addition to the overall score, has four measures: somatic symptoms; anxiety/insomnia; social dysfunction and severe depression symptoms. The severity of these negative mental conditions is measured by summing up the answers to specific questions, coded in a dichotomous system. Having considered the foregoing, total measures for individual domains can take values 0–7 points, and 0–28 points for total measures. The higher the GHQ value, the worse the mental health. These measures are standardized by the authors of the questionnaire. The original GHQ-28 and Polish version both have been extensively validated and both have clear scoring guidelines.

The Subjective Work Evaluation Questionnaire (SWEQ) by Dudek et al. was used for assessment of subjective work characteristics (1). This questionnaire is used to measure the subjective perception of work and is designed to measure employees' individual sense of professional stress. It consists of 50 statements describing different characteristics of work. These are numbered from 1 to 5 so as to indicate the extent to which a particular characteristic is onerous (1 - the characteristic is not present in the job, 5 - the highest degree of nuisance). The questionnaire has been extensively validated and has clear scoring guidelines.

### Statistical Methods

In the descriptive part, we have prepared the characteristics of the study population in the form of tables containing values of selected descriptive statistics for numerical characteristics or percentage distribution of selected characteristics.

The analysis of the relationship between two numerical (ordinal) characteristics was carried out by determining Spearman's coefficient of rank correlation (r_S_) and supplemented by the results of the significance test of the correlation coefficient *(p*).

We constructed six regression models in which the overall mental health measure was the dependent variable, and the set of independent variables consisted of SWEQ psychometric measures and selected demographic and professional factors of age, education and ward type. Using a progressive stepwise procedure, we then selected optimal model forms in which we took only statistically significant factors into account and interpreted them.

## Results

### Study Group

The research group consisted of 558 nurses. The vast majority of the respondents were women (92.5%). Nurses younger than 34 years constituted 44.8%, and those older than 51 years – 15.4% of the respondents. Over 40% of the respondents had a master's degree in nursing. Those with work experience shorter than 6 years constituted 35.5% and longer than 17 – 29.6% of all the respondents. The demographic characteristics of the analyzed group of nurses are presented in Table 1.

**Table 1:** Basic demographic characteristic.

Sex^a^	Female	516	92.5%
	Male	42	7.5%
Education^a^	No master's degree	332	59.5%
	Master's degree	226	40.5%
Age (years)^b^		37.3 ± 11.5	22–60
Work experience (years)^b^		11.8 ± 8.8	0–34

a *Counts and percent*.

b *Mean ± std. dev. & minimum-maximum range*.

### Mental Health of the Nurses

Table 2 presents statistics on the distribution of GHQ-28 measures in the entire study population. As can be seen, mental discomfort is manifested primarily by the presence of somatic symptoms (mean 2.26 points) and anxiety/insomnia (mean 2.15 points), with social dysfunction to a lesser extent, and severe depression being the least manifesting. It should be noted that there is a very significant asymmetry in the distribution of GHQ measures (apart from the overall measure) – the mean values are clearly higher than the medians. In case of social dysfunction and severe depression medians are 0 points, meaning that at least half of the nurses do not experience any mental problems in these areas.

**Table 2:** Mental health of the nurses.

**GHQ-28**	**Mean**	**Median**	**Std. dev**.	**Min**	**Max**
Somatic symptoms	2.26	2	2.28	0	7
Anxiety/insomnia	2.15	1	2.27	0	7
Social dysfunction	1.41	0	2.00	0	7
Severe depression	0.41	0	1.11	0	7
Total	6.23	5	6.35	0	28

### Subjective Evaluation of Negative Work Features

The subjective evaluation of negative features of nurses' work was carried out using the 50-position SWEQ questionnaire, based on which the score-based measures of workload in 10 selected aspects are calculated (1). These measures are pejorative in nature – their higher values mean worse result of work evaluation. Table 3 presents information on the distribution of numerical measures of work features.

**Table 3:** Subjective evaluation of negative work features.

**Subjective Work Evaluation Questionnaire**	**Mean**	**Median**	**Std. dev**.	**Min**	**Max**
General stress	129.7	128	35.5	55	233
Work overload	20.8	20	7.1	9	43
Lack of rewards	18.7	19	6.3	8	37
Uncertainty in workplace	17.8	18	5.7	7	35
Social relations	11.3	10	3.5	5	25
Threat	13.3	13	3.7	5	25
Physical burdens	9.8	8	5.3	4	20
Unpleasant work conditions	5.9	3	3.6	3	15
Lack of control	9.4	9	2.9	4	20
Lack of support	5.8	5	2.6	3	14
Responsibility	10.3	10	3.1	4	19

The constituent measures in the SWEQ questionnaire were not standardized by the questionnaire authors, thus making direct comparison impossible. Because of that, we have distinguished people who were classified as having a high level of negative work characteristics according to the thresholds established by the authors of the SWEQ questionnaire (1). Table 4 presents a summary of the numbers and percentages of people with a high level of negative assessments related to a given aspect of work. The results, apart from general stress, were ranked from the characteristics most often regarded as negative, to those rarely indicated by the respondents.

**Table 4:** Number and percentage of nurses with high levels of negative work characteristics.

**Nurses with high level of stress^a^**	***N***	**%**
General stress (>101 pts)	430	77.1%
Threat (>9 pts)	466	83.5%
Responsibility (>7 pts)	442	79.2%
Social relations (>8 pts)	441	79.0%
Lack of rewards (>13 pts)	423	75.8%
Lack of control (>7 pts)	386	69.2%
Uncertainty in workplace (>14 pts)	382	68.5%
Work overload (>16 pts)	371	66.5%
Physical burdens (>7 pts)	363	65.1%
Lack of support (>4 pts)	349	62.5%
Unpleasant work conditions (>4 pts)	253	45.3%

a *Thresholds of high level for each SWEQ measure are shown in parentheses*.

### Correlations Between the Assessment of Negative Work Features and Mental Health Measures

We then assessed the relationship between work stress measures and mental health measures by determining Spearman's rank correlation coefficients between GHQ-28 and SWEQ measures. The analysis covered the entire population.

Based on the correlation matrix presented in Table 5, it can be concluded that the relatively strongest correlations are between the total evaluation of stress at work and the total evaluation of mental health, as well as between *work overload* and total health evaluation (r_S_ = 0.39). Clearly the weakest correlations were found between *unpleasant work conditions* and total health measures (r_S_ = 0.12–0.15).

**Table 5:** Spearman correlation coefficients between the assessment of negative work characteristics and mental health measures.

**Subjective Work Evaluation Questionnaire**	**GHQ-28**				
	**Somatic symptoms**	**Anxiety/insomnia**	**Social dysfunction**	**Severe depression**	**Total**
General stress	0.29^***^	0.37^***^	0.37^***^	0.30^***^	0.39^***^
Work overload	0.29^***^	0.37^***^	0.37^***^	0.28^***^	0.39^***^
Lack of rewards	0.27^***^	0.31^***^	0.33^***^	0.25^***^	0.35^***^
Uncertainty in workplace	0.26^***^	0.31^***^	0.31^***^	0.26^***^	0.34^***^
Social relations	0.22^***^	0.27^***^	0.29^***^	0.28^***^	0.30^***^
Threat	0.19^***^	0.27^***^	0.29^***^	0.22^***^	0.28^***^
Physical burdens	0.18^***^	0.24^***^	0.22^***^	0.17^***^	0.24^***^
Unpleasant work conditions	0.12^**^	0.14^**^	0.12^**^	0.12^**^	0.15^***^
Lack of control	0.25^***^	0.33^***^	0.30^***^	0.25^***^	0.34^***^
Lack of support	0.22^***^	0.26^***^	0.29^***^	0.26^***^	0.30^***^
Responsibility	0.16^***^	0.20^***^	0.22^***^	0.24^***^	0.23^***^

Statistical significant dependencies:

** p < 0.01;

*** *p < 0.001*.

### The Effect of the Overall Measure of Work-Related Stress on Total Mental Health Measure, Including Occupational and Demographic Factors

Based on the correlation analysis presented in Table 5, we found that the general measure of stress SWEQ and total mental health measure GHQ-28 are statistically significantly related. However, in order to check if this is not an apparent dependence and to discover what factors influence the strength of this dependence, we conducted a regression analysis in which the dependent variable was the total measure of GHQ-28 and the independent variables were:

- SWEQ general measure of stress at work;
- age – in a dichotomous division into two groups (under the age of 40 and 40 or older);
- education (divided into secondary/bachelor degree vs. higher education);
- ward (Emergency, Internal and Surgical).

We selected age, education and ward type as occupational and demographic factors, because we found no statistically significant correlation between sex, place of residence and marital status of the respondents and any of the SWEQ and GHQ-28 questionnaire components.

In the first stage, we introduced all independent variables into the model, additionally taking into account the second degree interactions between them. There were a total of four main factors and six interactions in the model. Most of the factors in this model were statistically insignificant, so we decided to search for the optimal form of the model by means of a progressive stepwise regression procedure. The result is a fairly simple model with one main factor and one interaction. It turned out that age and ward do not affect total GHQ-28 measure statistically significantly, neither as separate factors nor in interactions. The model presented in Table 6 includes two factors: SWEQ general stress at work and education, but in interaction with the SWEQ general stress measure.

**Table 6:** The effect of the overall measure of work-related stress and education levels on overall mental health.

**Predictors**	**GHQ-28 (total)**		
	***R***^****2****^ **=** **18.7%** ***F*** **=** **13.5** ***p*** **=** 0.0000^***^		
	***B* (95% c.i.)**	***p***	**ß**
General stress at work (SWEQ)	0.073 (0.059; 0.087)	0.0000^***^	0.41
Education (higher vs. secondary) × general stress at work (SWEQ)	−0.006 (−0.009; −0.002)	0.0032^**^	−0.11

*R^2^, coefficient of determination; F, test statistic and p-value for significance of whole model; B, regression coefficient with 95% c.i., p-value for significance of each regression coefficient; ß, standardize regression coefficient*.

** p < 0.01;

*** *p < 0.001*.

The model explains a total of 18.7% variation of GHQ-28 in the study population. Detailed interpretation of model parameters is as follows:

- As the general measure of stress at work increases by 1 point, the total GHQ-28 increases, on average by about 0.073 point.
- The effect of the SWEQ general stress measure on the total GHQ-28 is education-dependent.

**Figure 1:**
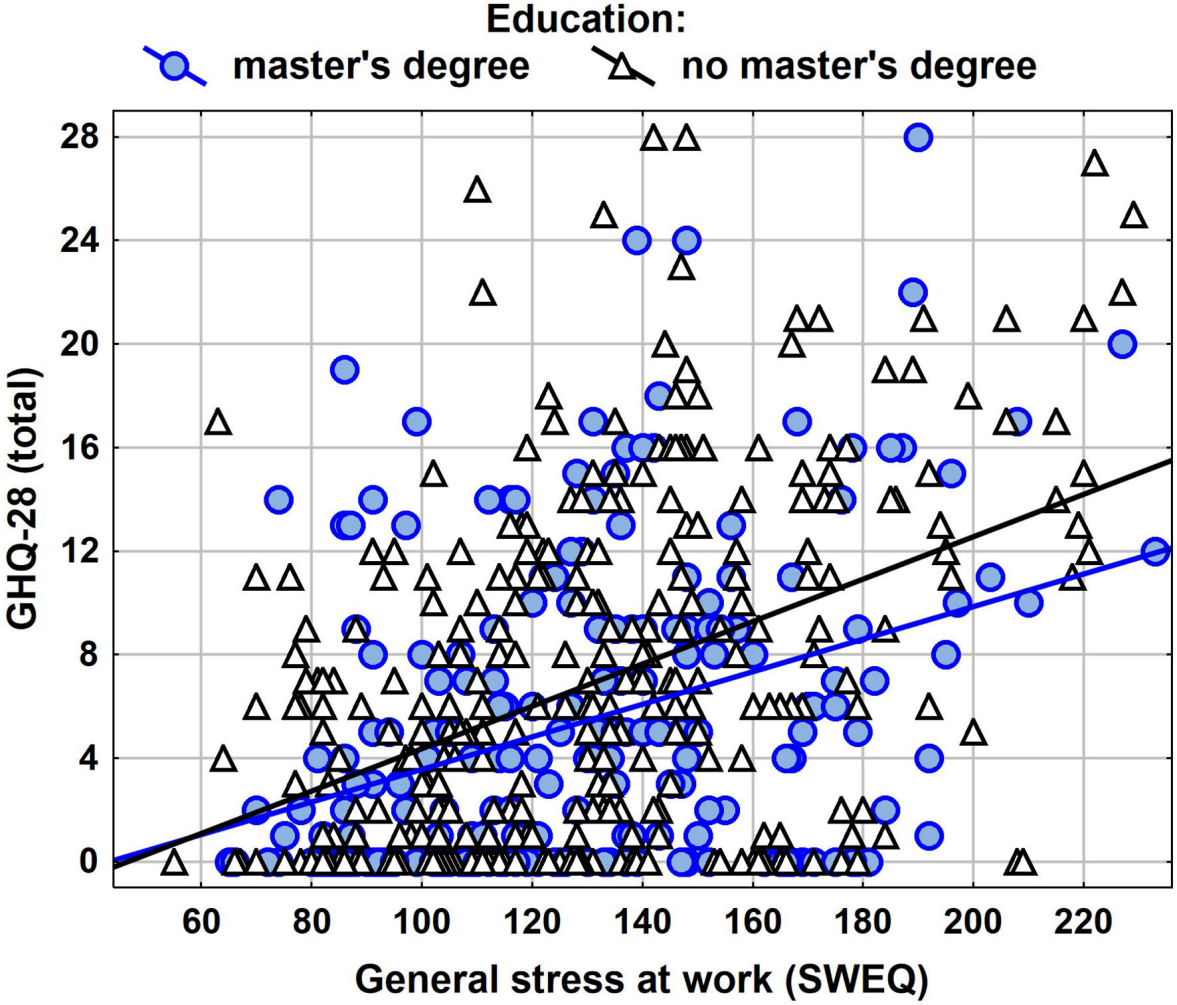
NImpact of SWEQ general measure in interaction with education level on GHQ-28 total.

Due to relatively simple regression model form, it is possible to illustrate the results in the form of a scatter plot (Figure 1). This shows both the effect of the SWEQ general measure and its correction for education level. Looking at the position of the simple regressions, we can see a stronger influence of the SWEQ general measure on the GHQ-28 total measure among nurses without higher education.

### The Effect of Partial Psychometric Measures on the Overall Mental Health Level

In the next analysis, we used regression analysis to assess which of the ten partial psychometric measures determined using the SWEQ questionnaire have a statistically significant effect on the overall GHQ-28 measure and which percentage of GHQ-28 variability can be explained when synthesizing information about the level of stress experienced at work.

The preliminary analysis of the correlation coefficients in Table 5 shows that all SWEQ components are statistically significantly related to the GHQ-28 total measure. Subsequently, we constructed a regression analysis model in which we adopted the total GHQ-28 as a dependent variable, and we introduced all 10 SWEQ partial measures as independent variables.

The full model, with all the SWEQ measures allowed explaining the variability of GHQ-28 mental health status to 19.2%. However, only two components: *work overload* and *responsibility*, were statistically significant *(p* < 0.05). A model in which as many as eight independent characteristics were statistically insignificant could not be the subject of final conclusions. Because of this, by applying a progressive stepwise regression procedure, we selected five SWEQ measures that were the most important determinants of the nurses' mental state: *work overload, lack of rewards, social relations, lack of support* and *responsibility*. Table 7 shows the model that was subject to final interpretation.

**Table 7:** The effect of partial psychometric measures SWEQ on the overall mental health level.

**Predictors**	**GHQ-28 (total)**		
	***R***^****2****^ **=** **19.2%** ***F*** **=** **25.9** ***p*** **=** 0.0000^***^		
	***B* (95% c.i.)**	***p***	**ß**
Work overload	0.202 (0.093; 0.312)	0.0003^***^	0.23
Lack of rewards	0.125 (0.005; 0.245)	0.0419^*^	0.12
Social relations	0.259 (0.052; 0.465)	0.0142^*^	0.14
Lack of support	0.267 (0.003; 0.530)	0.0472^*^	0.11
Responsibility	−0.241 (−0.464; −0.017)	0.0348^*^	−0.12

*R^2^, coefficient of determination; F, test statistic and p-value for significance of whole model; B, regression coefficient with 95% c.i., p-value for significance of each regression coefficient; ß, standardize regression coefficient*.

* *p < 0.05*;

*** *p < 0.001*.

Taking into account the standardized regression coefficient values ß, we found that the most important factor is *work overload*, while the importance of the others is similar. This model explains more than 19.2% of the variability of the GHQ-28 total measure.

### The Influence of Partial Psychometric Measures on the Overall Level of Mental Health, Including Occupational and Demographic Factors

The model developed in this section is an extension of the results presented in the model presented in Table 7, by introducing demographic and occupational variables as additional control factors. We introduced three factors (ward, education, age) and thirty interactions between each of these factors and the SWEQ partial measures. Then, using a progressive stepwise regression procedure, we determined statistically significant factors. The results are presented in Table 8.

**Table 8:** The effect of partial psychometric measures SWEQ and level of education on the overall mental health level.

**Predictors**	**GHQ-28 (total)**		
	***R***^****2****^ **=** **20.7%** ***F*** **=** **35.4** ***p*** **=** 0.0000^***^		
	***B* (95% c.i.)**	***p***	**ß**
Work overload	0.196 (0.104; 0.288)	0.0000^***^	0.22
Social relations	0.254 (0.060; 0.449)	0.0106^*^	0.14
Lack of support	0.291 (0.036; 0.547)	0.0252^*^	0.12
Education (higher vs. secondary) × unpleasant work condition	−0.149 (−0.220; −0.078)	0.0000^***^	−0.16

*R^2^, coefficient of determination, F, test statistic and p-value for significance of whole model; B, regression coefficient with 95% c.i., p-value for significance of each regression coefficient; ß, standardize regression coefficient*.

* *p < 0.05*;

*** *p < 0.001*.

As can be seen, the first three measures have values not too different from those in the model showed in Table 7. Absence of *lack of reward* and *responsibility* measure effect, and the emergence of a statistically significant interaction between education and the effect of *unpleasant work conditions* on the total measure of mental health are significant differences. A negative coefficient for the interaction effect means that the effect of the unpleasant working conditions factor on the total GHQ-28 measure is weaker among nurses with higher education and stronger among nurses with secondary education.

## Discussion

The aim of our study was to determine the effect of stress experienced by nurses on their mental health in interaction with occupational and demographic factors. Generally speaking, nurses consider their work to be highly stressful. This is reflected by the high position of nursing in rankings of the most stressful professions. According to studies conducted in Poland, nursing is in the second group of the most stressful occupations (3), and in the USA, nursing is in the top five most stressful occupations according to the scale developed by the Occupational Information Network (31).

In our survey, as many as 77.1% of all respondents described the perceived overall level of stress caused by work as very high. Some of the components of the SWEQ questionnaire were assessed as very high by an even higher percentage of respondents. *Threat* results in very high stress in 83.5% of all respondents, with *responsibility* and *social relations* inducing this ranked at 79.0 and 79.2%, respectively. Only *unpleasant work conditions* clearly differs from the other measures, as this was indicated as a highly stressful factor by 45.3% of all respondents. The fact that the percentage of results considered high is very high is due to the fact that the questionnaire authors set standards while taking different professions into account (1). This means that nurses see relatively many negative characteristics in their work compared to other professions. In similar studies carried out in hospitals in other parts of the country, the most common stressors were *responsibility, work overload* and *threat* (23, 32). Most of the studies conducted among nurses in different countries indicated *work overload* as the most stressful characteristic of work. In the case of the incidence of the remaining stressful characteristics in the nurses' work, no significant regularity was observed among the published study results (33–35). This is probably due to cultural differences and the use of different research tools, as there are no popular international questionnaires such as the GHQ series for subjective evaluation of work characteristics.

In general, nurses are quite satisfied with their mental health. At least ¼ of the respondents do not complain about any mental discomfort, and at least half of them do not show social dysfunction. As many as ¾ of all respondents do not experience the symptoms of depression. However, the distribution of GHQ-28 mental health measures in our studies is characterized by high asymmetry. This was clearly notable in the case of somatic symptoms and anxiety/insomnia where ¼ of the respondents assess their condition significantly worse than average. By selecting GHQ-28 score of 6 as the cut-off point, it can be concluded that 39.9% of all nurses suffer from mental disorders. Somatic symptoms were the most frequent and severe depression was the least frequent. In this respect, our results differ from the results obtained in the study conducted in Iran (36), where 45.4% of all nurses complained about mental disorders, but social dysfunctions were by far the most common disorder, and somatic symptoms were only ranked third. In studies carried out among Greek nurses, depressive conditions were much more frequent than in our country, whereas anxiety/insomnia were at similar level (37). In Lithuania, Poland's neighbor, as much as 60.4% of the surveyed nurses assessed their mental health as poor (38). Very similar results to ours were obtained in a study conducted in Poland in the neighboring Lublin Voivodeship, where 38.1% of all nurses suffered from mental disorders, and while severe depression was the least frequent, the remaining components were at a similar level (39).

Our work indicated that negative characteristics of the work are associated with the assessment of bad mood. The correlation matrix presented in Table 5 shows the existence of statistically significant correlations between all negative work characteristics (SWEQ) and measures of mental health (GHQ-28). All correlations are positive and highly statistically significant, with the exception of *unpleasant working conditions* and GHQ-28 questionnaire partial measures, which are only statistically significant. Similar relationships were obtained in studies conducted by other researchers (40, 41). Furthermore, very similar results were obtained in studies conducted in Japan (42). Indeed, even the strength of most correlations is almost identical.

The co-occurrence of negative work characteristics and poor well-being does not indicate how negative work characteristics can affect the mental health of nurses. Using regression models, we studied how the subjective overall measure of stress at work, demographic and occupational factors, separately and in interaction with each other, can influence the GHQ-28 total measure of nurses' mental health. We did not find any effect of age or ward type, alone or in interaction with the nurses' assessment of their mental condition. The final version of the model showed that only the SWEQ general measure of stress alone and in interaction with education affects the overall mental well-being of the GHQ-28 surveyed nurses. While the increase in the overall SWEQ stress at work measure results in worse overall psychological well-being of the subjects, after analyzing interactions with education, it can be concluded that nurses with a higher education level demonstrate better mental capacity to withstand workplace stress. Similar results were obtained in studies carried out in Silesia (23). However, while studies carried out in other countries confirm the negative effect of occupational stress on the mental health of nurses (43), the effect and interaction of other factors is different. For example, some researchers show interaction with the type of ward or seniority (9, 21, 44, 45).

Examination of the effect of the partial psychometric measures from the SWEQ questionnaire on the overall GHQ-28 measure showed that the most significant effect was demonstrated by *work overload*. *Lack of rewards, social relations, lack of support* and *responsibility* showed lower effect, and the other measures did not affect the GHQ-28 overall measure. The increase in stress caused by *work overload, lack of rewards, social relations* and *lack of support* caused deterioration in the mental well-being of nurses, whereas in the case of the *responsibility* measure, the coefficient was negative. This means that an increase in the level of stress induced by the sense of responsibility improves the psychological well-being of the studied nurses. This is an apparent paradox, since as many as 79.2% of all respondents described *responsibility* as a highly stressful factor, and it was the second most stressful factor in terms of frequency. After deeper analysis, we have come to the conclusion that an increase in the sense of responsibility alone, with no changes to the other SWEQ measures, can actually mean greater certainty for the nurse at work and have a positive effect on overall mental health. *Work overload, lack of rewards, social relations, lack of support* negative effect and the positive impact of increased sense of *responsibility* on the mental health of nurses have been presented in other studies (46–48). These results are in line with the theoretical Job Demand Control Support model (18) and Effort Imbalance Model (19).

The final part of the analysis was to examine the influence of partial psychometric measures in interaction with occupational and demographic factors on the overall GHQ-28 measurement. The constructed regression model again showed the strong effect of *work overload*, with the much weaker effect of *social relations* and *lack of support* on the deterioration of nurses' well-being, as measured by the overall measure in the GHQ-28 questionnaire. However, the most interesting result was the significant effect of education in interaction with *unpleasant work condition*. The negative coefficient for this interaction can be interpreted in such a way that nurses with higher education are more resistant to the negative effect of *unpleasant work* conditions on overall mental health self-assessment. The effect of higher education as a factor increasing the nurse resistance to stress at work and its positive effect on well-being self-assessment was confirmed by other researchers (49, 50). The special interaction between education and *unpleasant work conditions* resulting from our research deserves attention and can be a complement to early research.

Summarizing the above, the stress in the workplace determined by the general measure of the Subjective Work Evaluation Questionnaire has a negative effect on the self-assessment of the mental health of the nurses by means of the GHQ-28 questionnaire. Among the partial measures of the SWEQ questionnaire, *work overload* had strong effect, whereas the *lack of rewards, social relations* and *lack of support* had a weak negative effect on the overall mental health self-assessment of nurses. *Responsibility* was an exception that had a positive effect on the nurses' well-being. Among occupational and demographic factors, only higher education in relation to secondary education in interactions with the general measure of stress and *unpleasant work conditions* had a positive effect on the overall mental health self-assessment of nurses, determined using the GHQ-28 questionnaire.

## Conclusions

1. Stress caused by work overload is the factor most negatively influencing the self-assessment of mental health in nurses. A greater sense of responsibility for one's work has a positive effect on the self-assessment of the mental health in nurses.
2. Higher education in relation to secondary education is a factor that positively affects the self-assessment of mental health in nurses. Nurses with higher education show better tolerance concerning unpleasant work conditions as a stress factor negatively affecting their mental health.
3. The results of our study provide a clear message to the hospital management, consistent with other studies (29). Improving the organization and atmosphere at work of nurses toward reducing perceived work overload and increasing the responsibility of nurses can have a positive impact on the mental health of nurses. Encouraging nurses to improve their education can result not only in an obvious improvement in staff qualifications, but also in better resistance to stressors in the workplace and, consequently, in better staff well-being. Both measures can have a positive impact on the quality of care provided by nurses and can reduce staff turnover.

## Methodological Limitations

The sample used, study design (cross-sectional study), self-reported style questionnaires and are significant limitations of the study. The research was conducted only in a single region of Poland. The respondents were completing questionnaires remotely, which could lead to data bias and low response rate. The reasons for 31% of all invited persons not taking part in the study are unknown due to the manner the study was conducted.

## Data Availability Statement

The raw data supporting the conclusions of this article will be made available by the authors, without undue reservation.

## Ethics Statement

The studies involving human participants were reviewed and approved by Bioethical Commitee of Medical University of Bialystok, Poland. Written informed consent for participation was not required for this study in accordance with the national legislation and the institutional requirements.

## Author Contributions

KK: concept of the research, design of article structure, conducting of the research, review of the literature, results analysis, and writing the article. EK-K: review of the literature and review of article drafts. MS: statistical analysis. All authors contributed to the article and approved the submitted version.

## Conflict of Interest

The authors declare that the research was conducted in the absence of any commercial or financial relationships that could be construed as a potential conflict of interest.
